# Revealing the spatiotemporal evolution pattern and convergence law of agricultural land transfer in China

**DOI:** 10.1371/journal.pone.0300765

**Published:** 2024-06-06

**Authors:** Min Sheng, Wenting Shi, Xiaobiao Lin, Bowei Wu, Shidai Wu

**Affiliations:** 1 School of Culture Tourism and Public Administration, Fujian Normal University, Fuzhou, Fujian, P. R. China; 2 College of Sociology and History, Fujian Normal University, Fuzhou, Fujian, P. R. China; 3 School of Geography Sciences, Fujian Normal University, Fuzhou, Fujian, P. R. China; Qufu Normal University, CHINA

## Abstract

The transfer of land plays a crucial role in revitalizing land resources, acting as a catalyst for promoting the high-quality development of agriculture. The land transfer ratio is a crucial metric for assessing the progress of rural land transfer and the effective allocation of rural land resources. Thus, this study examines the rural land transfer ratio across 30 provinces in China from 2005 to 2020. The study explores the distribution characteristics of the ratio using the rank-size rule and trend surface analysis. The LISA space-time transition method is employed to analyze the spatial and temporal dynamics of the rural land transfer ratio and examine its convergence. The study aims to comprehensively analyze the spatial distribution characteristics and evolutionary patterns of rural land transfer in China, illustrating the convergence and influencing factors during the development process. The results indicate that: (1) The rural land transfer ratio in China is generally increasing, with a spatial pattern showing an upward trend from west to east and from north to south. The main spatial contrast is between the eastern and western regions, with a relatively minor distinction between the southern and northern regions. (2) The LISA space-time transition highlights a significant spatial locking effect in China’s rural land transfer ratio, suggesting strong spatial integration in its evolution. (3) Clear indications of σ convergence, absolute β convergence, and club convergence are evident in China’s rural land transfer ratio. This suggests a gradual reduction in internal disparities among provinces and regions, where areas with higher land transfer ratios influence spatial spillover effects on adjacent lower areas. (4) Factors such as transportation infrastructure, irrigation, water conservancy construction, and farmers’ per capita income collectively influence the spatial and temporal evolution of China’s rural land transfer ratio, with dominant driving factors varying across different periods.

## Introduction

Amidst China’s ongoing urbanization and the restructuring of its rural industries, challenges such as farmland abandonment, insufficient rural land utilization, and the gradual decline of rural areas have gained increasing attention [1–3]. Land transfer refers to the transfer of land management and usage rights from farmers with land contractual management rights to protect their contractual entitlements [4]. As an integral component of China’s rural land system reform [5], land transfer covers multiple dimensions, including land ownership, contracting rights, and management rights. This process requires clear property rights and smooth circulation. Through systematic circulation of rural land management rights, the allocation of land production factors continuously optimizes, promoting the achievement of agricultural production scale, improving the efficient and intensive utilization of China’s rural land resources, and facilitating modernization. This, in turn, contributes to the sustainable development of China’s rural industry. The progression of rural land transfer in China unfolds across four stages. The initial phase, from 1978 to 1983, was characterized by a prohibition. In the early years of the reform and opening-up era, the Constitution of the People’s Republic of China explicitly prohibited the transfer of land in rural areas. In the exploratory phase from 1984 to 2001, China formally established the legal framework for land transfer with the enactment of the 1988 Amendment to the Constitution of the People’s Republic of China. Throughout this period, rural land transfer in China remained relatively subdued. The third phase, from 2002 to 2013, was marked by the promotion of institutionalization. In 2002, China enacted the Law of the People’s Republic of China on Land Contracting in Rural Areas, initiating a phase of institutionalization, proceduralization, and standardization in the administration of rural land transfer. Throughout this period, the scale of land transfer experienced a significant and rapid growth trend. In the current policy maturity period from 2014 onwards, rural China has formally entered the era of the "separation of powers ". The ownership rights, contracting rights, and management rights of agricultural land are distinctly managed, aiming to significantly enhance the circulation and management scale of agricultural land. This approach addresses the challenges of agricultural development more effectively, accelerates the transition from an agricultural power to an agricultural powerhouse, and ensures the preservation of land rights and interests for farmers.

Since the initiation of reforms and opening up, there has been continuous improvement in China’s rural labor mobility [4], the way agricultural land resource is used has been transforming from high-carbon to low-carbon [6], coupled with a gradual rise in the demand for rural land transfer. The Chinese government has proactively reformed the rural land system, promoting land transfer policies and providing incentives for farmers to participate in land transfer activities. China’s rural land transfer demonstrates a rapid growth trend, playing a crucial role in the sustainable modernization of Chinese agriculture.

Temporal and spatial changes in rural land transfer inevitably result in the reconfiguration and interaction of various factors, significantly influencing the rural economy. An examination of the theoretical foundation of land transfer and the marketization of property rights allocation in Marx’s theory of land property rights provides valuable guidance for the development of China’s farmland property rights system. Marx argues that land property rights are fundamental to the agricultural social and economic system [7]. Clearly defined land property rights are crucial for the orderly functioning of agricultural societies. Farmland transfer through market transactions essentially involves the transfer of property rights, treating land property rights as tradable commodities based on market value. In essence, land property rights should undergo commercialization, and allocation should follow market-oriented principles. Alchian A and Demsetz H explore the stability of land ownership, emphasizing its significance for the long-term investment of landowners. Limited rights diminish investment incentives and stability [8]. Coase R argues that a clear definition of property rights allows both parties to maintain reasonable expectations of their interests during transactions, facilitating the effective allocation of resources. A transparent property rights system is essential for reducing transaction costs and efficiently allocating resources. Coase’s theory of the property rights system sheds light on the connection between the structure of the rural land property rights system and the efficiency of rural economic operations [9]. These theories lay a theoretical foundation for the practice of rural land transfer and offer significant guidance for establishing the legal framework for rural land transfer.

With the advancement of research on rural land transfer, the analysis of rural land transfer modes has increasingly become a central focus. In both developing and developed nations, agricultural land leasing is widely acknowledged as the primary and effective method of land transaction [10–12]. According to Dong X, land leasing is the preferred transaction method among local farmers in various countries [10]. Initially, Basu’s research suggests that farmland leasing is the primary and efficient approach to land transactions in most developing countries, especially amid uncertainties in agricultural economic output [11]. Moreover, in developed nations, farmland leasing continues to be the dominant method of land transactions. Illustrated by Wegren’s study on rural land transactions, Russia serves as a case study for developed countries. The findings reveal that, in Russia, farmland leasing constitutes a substantial share of rural land transactions [12]. Research on factors influencing land transfer primarily focuses on the property rights system, transportation accessibility, family income status, agricultural machinery level, and other relevant factors [13–16]. Research on factors influencing land transfer primarily focuses on the property rights system, transportation accessibility, family income status, agricultural machinery level, and other relevant factors [13–16]. Firstly, a clearly defined rural land property rights system optimizes the allocation of land resources, reduces land transaction costs, and standardizes the rural land transfer process. Feder and Feeney clarify that land resources with well-defined property rights significantly contribute to enhancing agricultural investment and productivity. The clarification of land property rights reduces transaction costs, enabling large-scale operations by allocating production factors to the most efficient farmers, ultimately increasing agricultural productivity [17]. Regarding collective ownership of rural land in China, Kung K observes, through an examination of China’s non-agricultural labor market and rural land leasing market, that factors such as farmers’ age, family population, and education level will influence their willingness to transfer land [18]. Expanding on this, Deininger K argues that factors such as family per capita arable land area, family agricultural income, and degree of experience will also impact farmers’ land transfer decisions. Generally, farmers with non-agricultural income are more inclined to transfer their land [19].

China’s rural land transfer has attracted considerable attention from the academic community due to its crucial role in the development of China’s agricultural economy. Historically, numerous studies have predominantly used normative and literature analysis methods, focusing on the influencing factors and circulation methods of rural land transfer in specific provinces. However, there is a need to broaden and enhance the research perspective and foundational theory of land transfer. Moreover, considering the diverse development statuses and cultural backgrounds across different Chinese provinces, significant regional variations in the extent of land transfer are inevitable. Will these differences gradually diminish over time, displaying a trend toward convergence? Clearly, this issue is of great significance in understanding the ratio of rural land transfer and fostering the steady development of China’s agriculture amid the backdrop of rural revitalization.

Expanding on this foundation, the paper aims to challenge the analytical approach of current research and seeks to seamlessly integrate time and space, local and global elements, and solidification and flow cohesively from a geographical perspective. Utilizing spatial and economic geography methods, such as LISA space-time transition, convergence tests, and spatial and temporal geographical weighting, the paper thoroughly examines the spatial distribution characteristics and pattern evolution of rural land transfer in China. Additionally, it aims to illustrate the convergence of rural land transfer and the influencing factors in the development process. In doing so, it broadens the research perspective on land transfer, providing a theoretical and practical foundation, serving as a reference for decision-making in the study of rural land transfer policies in China within the context of rural revitalization. This paper makes a significant academic contribution by examining the spatiotemporal evolution pattern and driving factors of rural land transfer in China, a prominent global agricultural power. The study aids in scientifically discerning the laws and constraints governing the development and evolution of rural land transfer in China. Simultaneously, this research is of paramount importance in augmenting the efficacy of rural land resource allocation in China. It contributes to the intensification of rural land utilization and advocates for enhancing scale, efficiency, and modernization within the agricultural sector in China.

## Data sources, research objects, and methods

### Research objects and data sources

This research focuses on the remaining 30 provinces in China, excluding Tibet and Taiwan. The research data is obtained from the "China Rural Cooperative Economy Statistical Annual Report" and the "China Rural Policy and Reform Statistical Annual Report" covering the period from 2005 to 2020. For provincial categorization, the 30 provinces are classified into three regions—eastern, western, and central—based on the social and economic development of distinct areas in China [20–22]. Data collation, extraction, classification, and merging are performed based on the social and economic development of different regions in China. This compilation serves as the primary data source for the study.

### Selection of driving factors

**Fig 1:**
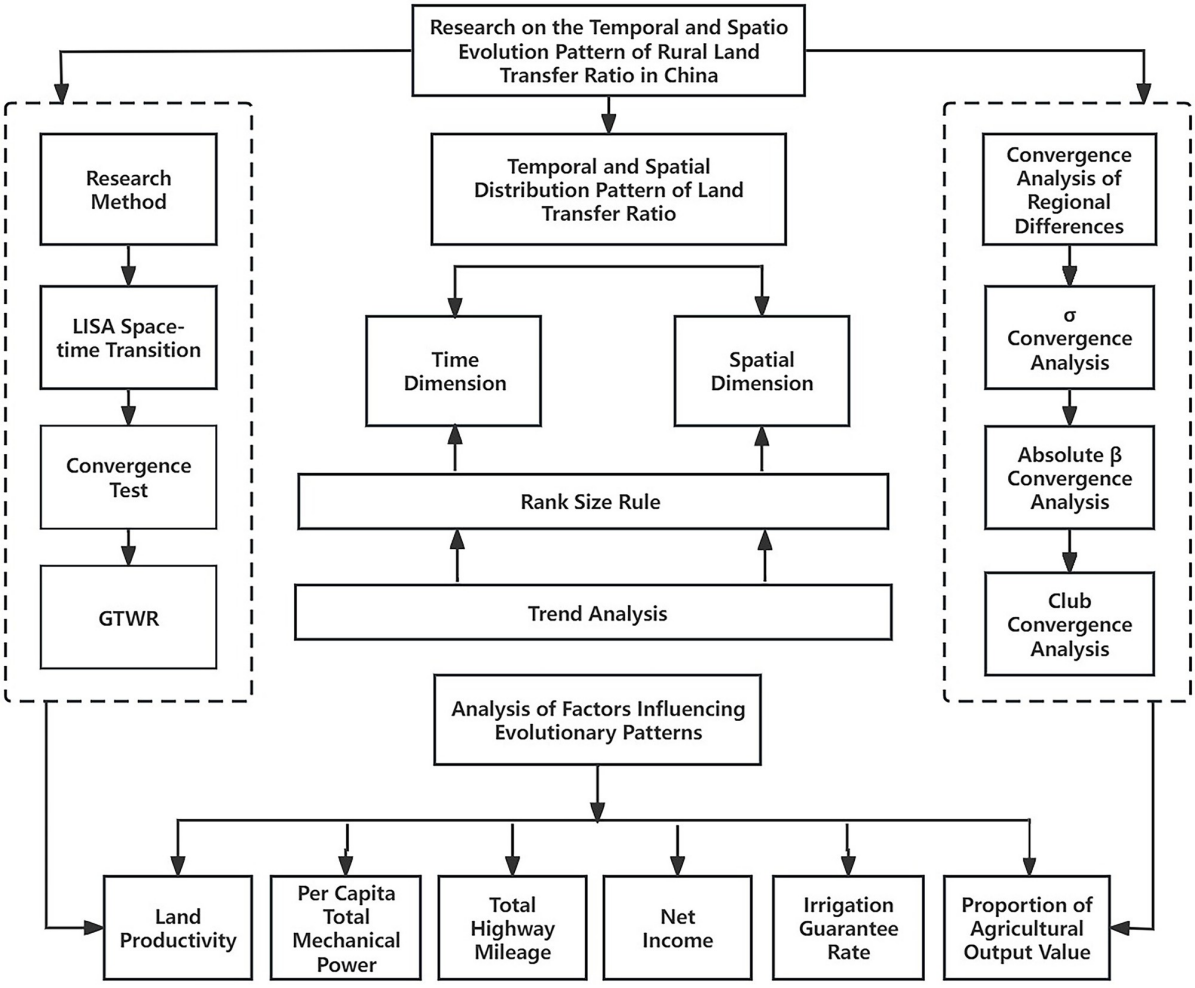
The conceptual framework of the primary research methods in this article.

Building upon Marx’s theory of property rights allocation marketization, land capitalization theory, and relevant economic geography theories, this study integrates the established spatio-temporal geographically weighted regression model with research on farmland transfer. Illustrated in Fig 1, the study selects explanatory variables, including land productivity, per capita total mechanical power, total highway mileage, per capita net income of farmers, irrigation guarantee rate, and agricultural output value, to establish a GTWR model. The study examines the temporal and spatial evolution pattern of rural land transfer in China and the influencing factors behind this transfer. The statistical descriptions of all major variables are presented in Table 1.

**Table 1:** Statistical description of main variables.

Variable	Observations	Mean	SD	Min	Max
**land Transfer Ratio**	480	0.2232	0.1264	0.0457	0.3698
**Land Productivity** **(10,000CNY/hm** ^**2**^ **)**	480	0.5845	0.3657	0.2280	1.6780
**Per Capita Mechanical Power(MW)**	480	6.0556	2.6345	2.2730	12.5930
**Total Highway Mileage(Km)**	480	139418.0420	81866.8150	4458.0000	285054.3000
**Per Capita Income of Farmers(CNY)**	480	33038.8108	13115.5016	9949.2627	68314.3809
**Irrigation Guarantee Rate(%)**	480	0.2871	0.1754	0.0543	0.8896
**The Proportion of Agricultural Output Value(%)**	480	0.0971	0.0543	0.0028	0.2527

### Research methods

Drawing on insights from academic research on Chinese rural land transfer, the study utilizes LISA space-time transition, a convergence test, and GTWR (Geographical and Temporal Weighted Regression) to explore the spatial distribution characteristics and pattern evolution of rural land transfer in China (as shown in Fig 1). The study also seeks to illustrate the convergence of rural land transfer and its influencing factors during the development process.

#### LISA space-time transition

The LISA space-time transition integrates the temporal dimension into the conventional static LISA, enabling dynamic and continuous spatial expression. This method is commonly applied to reveal the degree, direction, competition, and cooperation in spatial-temporal interactions among regional geographical elements. Additionally, it examines the impact of spatial-temporal dependence on the evolution of regional systems [23]. Key indicators of LISA space-time transition include path length, curvature, and direction of movement. Path length signifies the dynamic characteristics of the local spatial structure of China’s rural land transfer ratio. Curvature indicates the volatility of the local spatial structure of China’s rural land transfer ratio [24]. The direction of movement focuses on the integrated characteristics of the local spatial structure evolution of China’s rural land transfer ratio. The calculation formula is as follows:

$$d_{i}=N\sum_{t=1}^{T-1}d(L_{i,t},L_{i,t+1})/\sum_{i=1}^{N}\sum_{t=1}^{T-1}d(L_{i,t},L_{i,t+1})$$ (1)

$$\varepsilon_{i}=\sum_{t=1}^{T-1}d(L_{i,t},L_{i,t+1})/d(L_{i,t},L_{i,T})$$ (2)

$$\theta_{i}=arctan(\sum_{j}sin\;\theta_{j}/\sum_{j}cos\;\theta_{j})$$ (3)

In the formula, the path length and curvature of province i respectively denote the variation in the rural land transfer ratio within the province. A larger path length and curvature indicate a more pronounced change in the ratio. Likewise, larger changes in the local spatial structure result in a more complex representation of the rural land transfer ratio in the province. In the formula, N represents the number of provinces, T denotes the duration of the research stage, L_i_,t+1 signifies the LISA coordinate of province i at time t, and d(L_i_,t,L_i_,t+1) indicates the moving distance of province i from time t to t+1. Lastly, signifies the average moving direction of province i.

#### Convergence test

Drawing on insights from LISA space-time transition, which clarifies the spatial correlation degree and dynamic evolution of rural land transfer in China, this study introduces a convergence model to precisely depict the spatial heterogeneity development trend of the rural land transfer ratio across different provinces in China. The convergence of China’s rural land transfer ratio signifies the gradual reduction of regional differences in the rural land transfer ratio over time. Convergence analysis utilizes measurement methods such as σ convergence, β convergence (including absolute β convergence and conditional β convergence), and club convergence [25–27].

σ convergence. σ convergence refers to the trend where the disparity in land transfer ratios between regions diminishes over time. The coefficient of variation quantifies σ convergence, indicating the gradual decrease in regional land transfer ratio variation. The formula below is employed to calculate σ convergence [28]:

$$CV=\sqrt{\frac{1}{n}\sum_{i=1}^{n}{\left(LTR_{i}-\frac{1}{n}\sum_{i=1}^{n}LTR_{i}\right)}^{2}}/\left(\frac{1}{n}\sum_{i=1}^{n}LTR_{i}\right)$$ (4)

In the formula, CV denotes the coefficient of variation, the land transfer ratio of province i, and n signifies the number of research units.

β convergence. β convergence, classified into absolute β convergence and conditional β convergence, is investigated. Absolute β convergence refers to the situation where provinces with lower land transfer ratios undergo higher growth rates than provinces with higher land transfer ratios. Consequently, all provinces eventually reach the same land transfer ratio level. This reveals a negative correlation between the growth rate of the land transfer ratio and its initial level. This correlation leads to the attainment of an equilibrium steady state of the land transfer ratio in the region [29]. The formula is as follows:

$$In(LTR_{i,t+T}/LTR_{i,t})=\alpha +\beta In\left(LTR_{i,t}\right)+h_{i}+k_{t}+\varepsilon_{i,t}$$ (5)

In the formula, *In* (*LTR*_*i*,*t*+*T*_/*LTR*_*i*,*t*_) denotes the growth rate of province i’s LTR from year t to t+T; *LTR*_*i*,*t*_ is the LTR of the i-th province at the beginning of the study period; *LTR*_*i*,*t*_, is the LTR of the i-th province in period “t”; *α*, *β* are parameters to be estimated; T is the time span; *h*_*i*_, *k*_*t*_ are the individual effects that do not vary over time and the time effects that do not vary over time, respectively; *ε*_*i*,*t*_ is the random error term. Convergence speed θ = -ln(*β*+1)/T, if *β* is less than 0 and significant, then conditional convergence is considered to exist.

Club convergence. Acknowledging spatial heterogeneity, several international scholars have introduced the concept and definition of club convergence [30–32]. This study defines club convergence of the land transfer ratio as follows: provinces with the same or similar initial land transfer ratios exhibit comparable structural characteristics and tend to converge towards similar local stability. Variations in convergence exist among different club types. When confirming the presence of club convergence, the study is usually segmented into groups based on different regions or data characteristics. Within each group, verification utilizes absolute β convergence [33]. However, it is crucial to confirm the presence of absolute β convergence in the population where the group is located before conducting the verification. Therefore, this study initially verifies whether there is absolute β convergence in the land transfer ratio at the provincial level in China. Subsequently, each province in China is categorized into the eastern, western, and central regions to assess whether there is club convergence within each region.

#### GTWR

In previous studies examining spatial heterogeneity, the Geographically Weighted Regression (GWR) model was commonly utilized. While this model integrated spatial geographic location information into the regression equation to investigate the non-stationary nature of spatial relationships by adjusting parameter estimates based on spatial geographic location, it overlooked the temporal dimension and the issue of regression coefficients changing over time. To address this limitation, the Geographically Weighted Regression (GTWR) model integrates both time and space, resolving the issue of spatiotemporal instability and enhancing the relevance and effectiveness of the model’s estimation results. The fundamental formula is as follows [34]:

$$Y_{i}=\beta_{0}\left(\mu_{i},v_{i},t_{i}\right)+\sum_{k}\beta_{i}\left(\mu_{i},v_{i},t_{i}\right)X_{it}+\varepsilon_{i}$$ (7)

In the formula: (μ_i_, v_i_, t_i_) denotes the space-time coordinates of the i-th sample point, with longitude, latitude, and time individually represented as (μ_i_, v_i_, t_i_); *β*_0_ (μ_i_, v_i_, t_i_) signifies the regression constant of the i-th sample point, serving as the constant term in the model; *X*_*it*_ represents the value of the k-th independent variable at point i; *ε*_*i*_ is the residual; *β*_*i*_ (μ_i_, v_i_, t_i_) denotes the k-th regression parameter of the i-th sample point. The estimation method is as follows:

$$\hat{\beta }\left(\mu_{i},v_{i},t_{i}\right)={\left[X^{t}W\left(\mu_{i},v_{i},t_{i}\right)X\right]}^{-1}X^{T}W\left(\mu_{i},v_{i},t_{i}\right)Y$$ (8)

In the formula, $\hat{\beta }(\mu_{i},v_{i},t_{i})$ denotes the estimated value of β_k_ (μ_i_, v_i_, t_i_); X represents the matrix of independent variables; *X*^*t*^ signifies the transpose of the matrix; y denotes the matrix formed in the sample; W(μ_i_, v_i_, t_i_) represents the space-time weight matrix. W employs the Gaussian distance function and employs the bi-square space weight function to acquire the spatio-temporal weight matrix. The spatio-temporal distance between sample i and sample j is calculated as follows:

$$d_{ij}=\sqrt{\delta \left[{\left(U_{i}-u_{j}\right)}^{2}+{\left(v_{i}-v_{j}\right)}^{2}+\mu {\left(t_{i}-t_{j}\right)}^{2}\right]}$$ (9)

The selection of bandwidth significantly influences the formulation of space-time weight. The study employs the AICc rule and adaptive bandwidth.

## Spatial-temporal distribution pattern and evolutionary characteristics of rural land transfer ratios in China

### Overall distribution characteristics

#### Rank-size analysis

**Fig 2:**
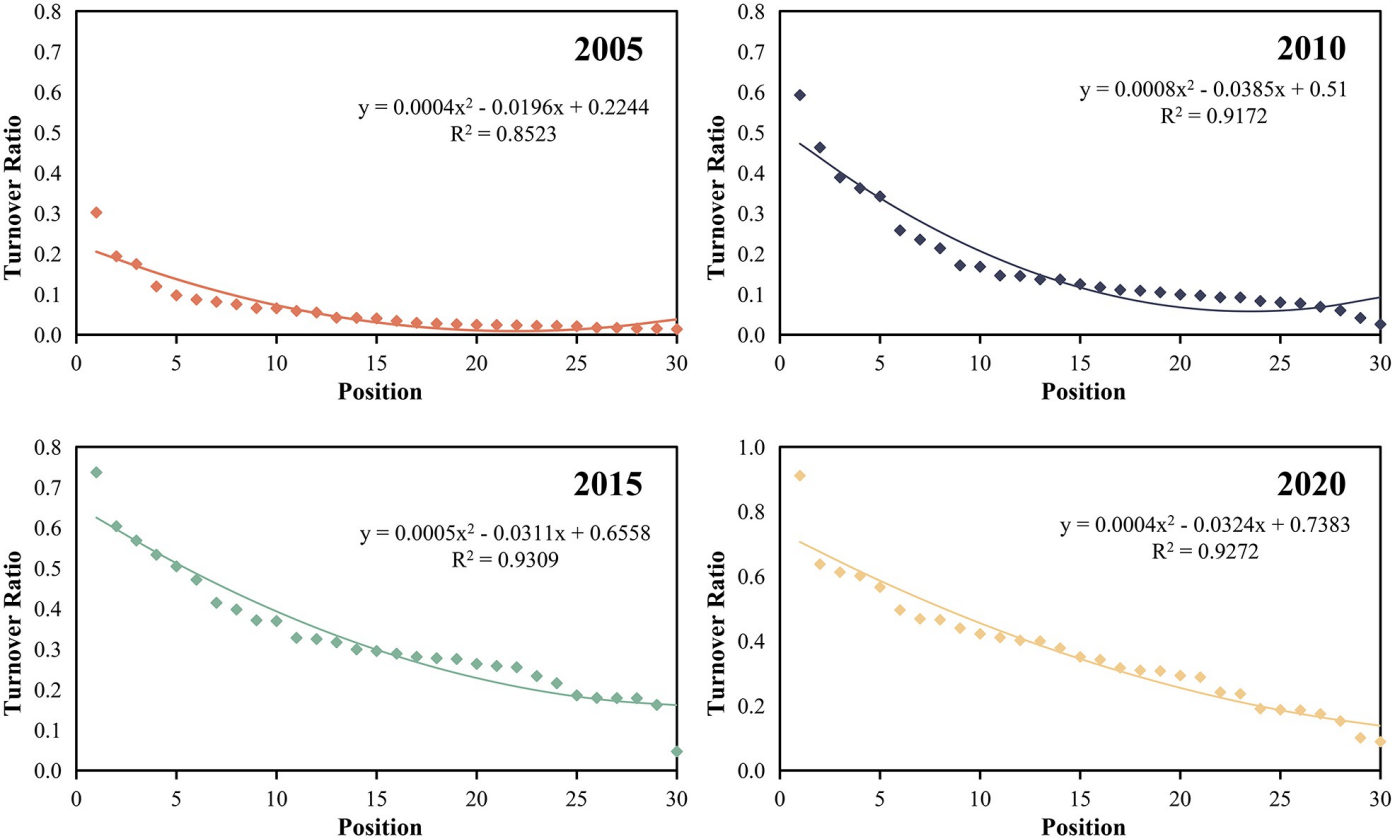
Rank-size analysis of rural land transfer ratio in China from 2005 to 2020.

Using collected data on rural land transfer area and total land area in China, the land transfer ratio for provincial units is computed annually. Calculating the regression curve between the land transfer ratio value and its rank, following the rank-size rule [35] (As shown in Fig 2). The goodness of fit (R^2^) for the regression curve exhibits a gradual upward trend, signifying an improved fitting effect. In 2005, 2010, 2015, and 2020, China’s rural land transfer ratio adhered to a numerical-rank distribution, where the top provinces had markedly higher ratios than the lower provinces. Put differently, the rural land concept and family characteristics of the top provinces are closely linked to factors such as the level of regional economic development, resource endowment conditions, land rights, social public service system, social security and land transfer mechanism, as well as government policies, laws, and regulations. The absolute value of the slope of the regression equation is less than 1, signifying a relatively small number of districts and provinces with high land transfer ratios. Counties with a relatively large middle-order land transfer ratio display a balanced degree of aggregation among provinces. This suggests that the land transfer ratio system in various provinces of China has reached a relatively mature stage. The absolute value of the slope of the regression equation exhibits a gradual increase, indicating that the trend of decentralization of the land transfer ratio in China’s provinces is less pronounced than the trend of concentration. The land transfer ratio of provinces in the middle rank is experiencing rapid growth, indicating a gradual shift towards a more reasonable land transfer ratio system in China’s provinces.

#### Overall trend analysis

**Fig 3:**
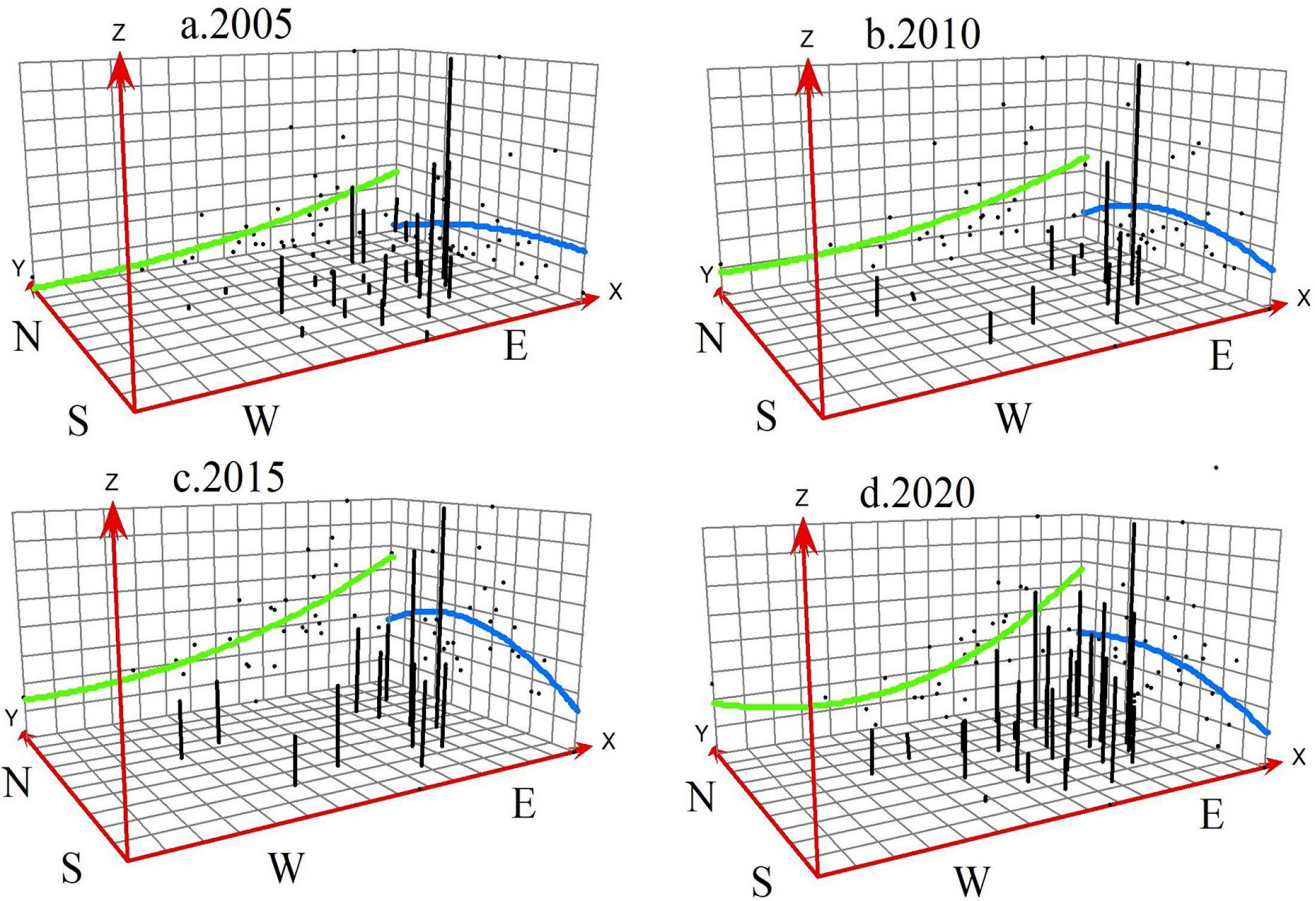
Trend surface analysis of rural land transfer ratio in China from 2005 to 2020.

To analyze the spatial distribution pattern and evolutionary context of China’s rural land transfer ratio from 2005 to 2020, this study utilizes trend analysis within the ArcGIS statistical tool, producing trend change maps for rural land transfer in the 30 provinces of China in 2005, 2010, 2015, and 2020 (As shown in Fig 3).

The analysis shows a spatial distribution trend in China’s rural land transfer ratio, with it being "high in the east and low in the west, high in the south and low in the north" across the four time intervals. Fig 3 shows that China’s rural land transfer ratio has an ascending trend from west to east along the X-axis and from north to south along the Y-axis. Over time, the disparities between the eastern and western regions are gradually diminishing, and the distinctions between the southern and northern regions are also reducing gradually. Ultimately, the circulation ratio between the southern and northern regions tends to reach equilibrium. This trend indicates that the pace of rural land transfer in the northern region is significantly higher than in the southern region, while the land transfer ratio in the central region is continuously evolving, with a rapidly improving speed of rural land transfer.

The study uncovers that the spatial disparity in rural land transfer ratios is intricately linked to various factors, including provincial land ideologies, family attributes, regional economic development levels, resource endowments, land ownership structures, social public service systems, social security measures, land transfer mechanisms, and governmental policies and regulations. Since the initiation of reform and opening up, Southern China has consistently experienced higher economic development than the northern regions, coupled with greater regional openness. The southern regions exhibit a more advanced and established social security and land transfer mechanism, leading to a higher rural land transfer ratio compared to the less developed northern regions. Additionally, to meet the practical needs of China’s social development and the requirements for intensive agricultural management, the government has implemented policies to encourage the consolidation of rural contracted land among farmers with agricultural expertise, aiming to promote intensive management and facilitate the development of moderately sized professional growers. However, with the acceleration of China’s urbanization and continuous economic development improvements, social security and land transfer mechanisms in Northern China are constantly improving. Additionally, the land ideologies and family characteristics of farmers have a significant influence on the rural land transfer ratio. The rural land transfer landscape is dynamic, with a notable acceleration in land transfer pace in Northern China. Consequently, the overall discrepancy in rural land transfer ratios between the North and the South is diminishing, attributed to advancements in economic development, social security measures, and land transfer mechanisms.

Generally, China’s rural land transfer ratio displays a more pronounced disparity between the eastern and western regions than between the northern and southern regions. This suggests that the spatial variation in the rural land transfer ratio is predominantly influenced by the contrast between the eastern and western regions, while the difference between the northern and southern regions is relatively minor. It is anticipated that the disparity between the eastern and western regions will further diminish beyond 2020.

### Spatial distribution pattern of rural land transfer ratio in China

**Fig 4:**
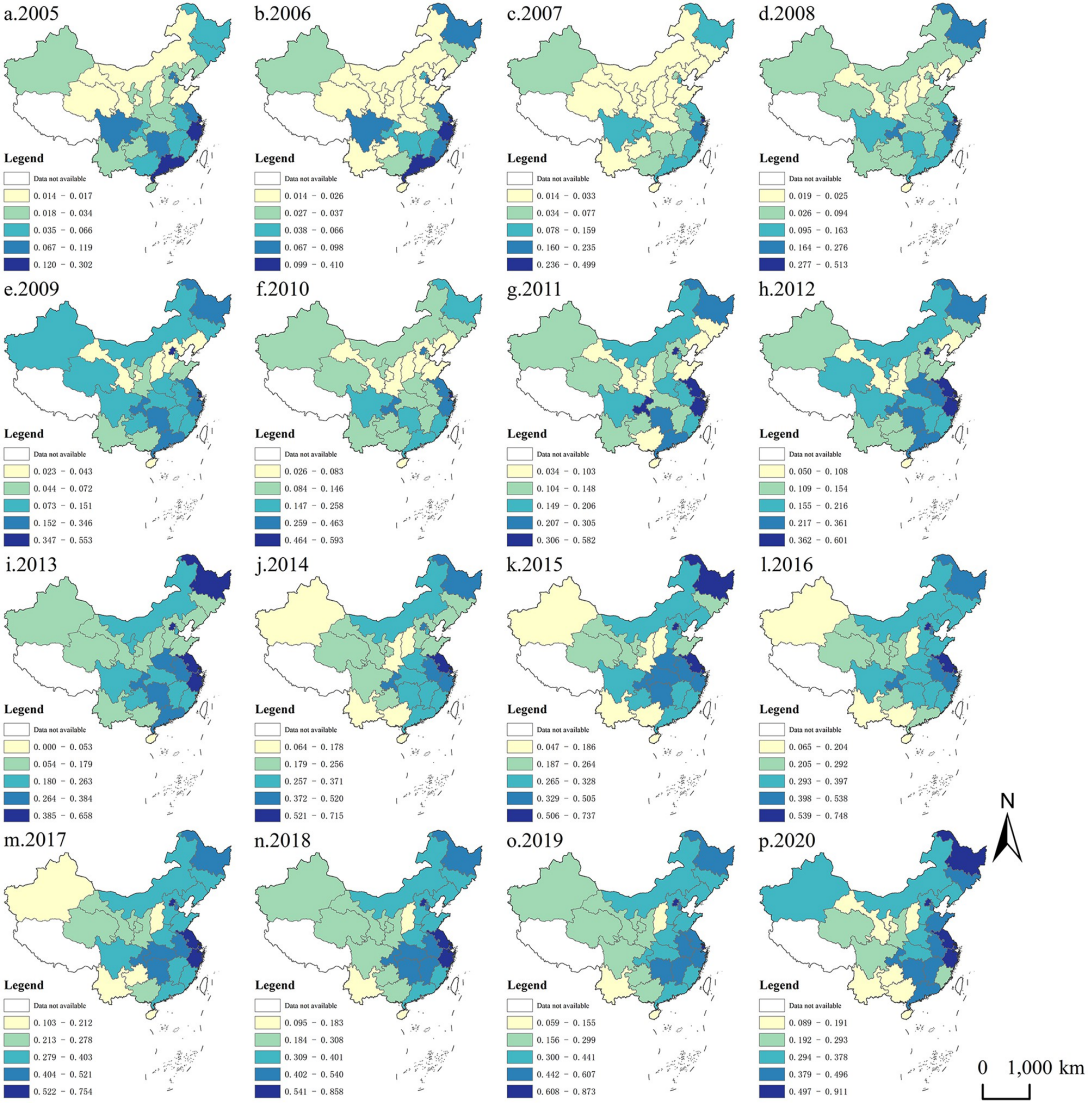
Spatial distribution pattern of rural land transfer ratio in China from 2005 to 2020.

Using statistical data on China’s rural land transfer ratio in the eastern, western, and central regions, this study visually represented the spatial distribution of the ratio from 2005 to 2020 through ArcGIS software (as depicted in Fig 4). The data categorizes China’s rural land transfer ratio into five classifications: (1) Provinces with a low circulation ratio (not exceeding 0.200); (2) Provinces with a low-medium circulation ratio (between 0.200 and 0.400); (3) Provinces with a medium circulation ratio (between 0.400 and 0.600); (4) Provinces with a higher circulation ratio (between 0.600 and 0.800); (5) Provinces with a high circulation ratio (exceeding 0.800).

Fig 4 illustrates the distinctive spatial distribution characteristics of China’s rural land transfer ratio, marked by elevated values in the east and south and lower values in the west and north. In 2005, heightened values were concentrated in eastern coastal provinces such as Guangdong, Zhejiang, and Jiangsu. Subsequently, by 2020, these high-value areas extended into the central and eastern regions. In contrast, low-value areas have concentrated in the western region since 2005, particularly in specific provinces in the northwest and southwest. Prominent high-value land transfers have emerged in the eastern region. In 2010, aligning with the strategy to boost central China, the concentration of land transfer ratios shifted gradually toward the eastern and central regions, predominantly the eastern region. By 2015, concurrent with the strategy to revitalize the old industrial base in Northeast China, the concentration of high-value land transfer ratios shifted towards Northeast China, particularly in Heilongjiang. In 2020, while the majority of high-value farmland transfer ratios are concentrated in the central and eastern regions, the western development strategy has caused certain provinces in the western region to become concentration areas for high-value transfer ratios. The comparatively lower farmland transfer ratio in the western region can be attributed to its lower level of economic development. In contrast to the eastern region, the western region experiences a distinct level of marketization for farmland transfer, along with variations in agricultural water conservancy facilities and transportation infrastructure, resulting in a noticeable gap between the two regions.

### LISA space-time transition analysis

#### LISA space-time transition geometric characteristics analysis

**Fig 5:**
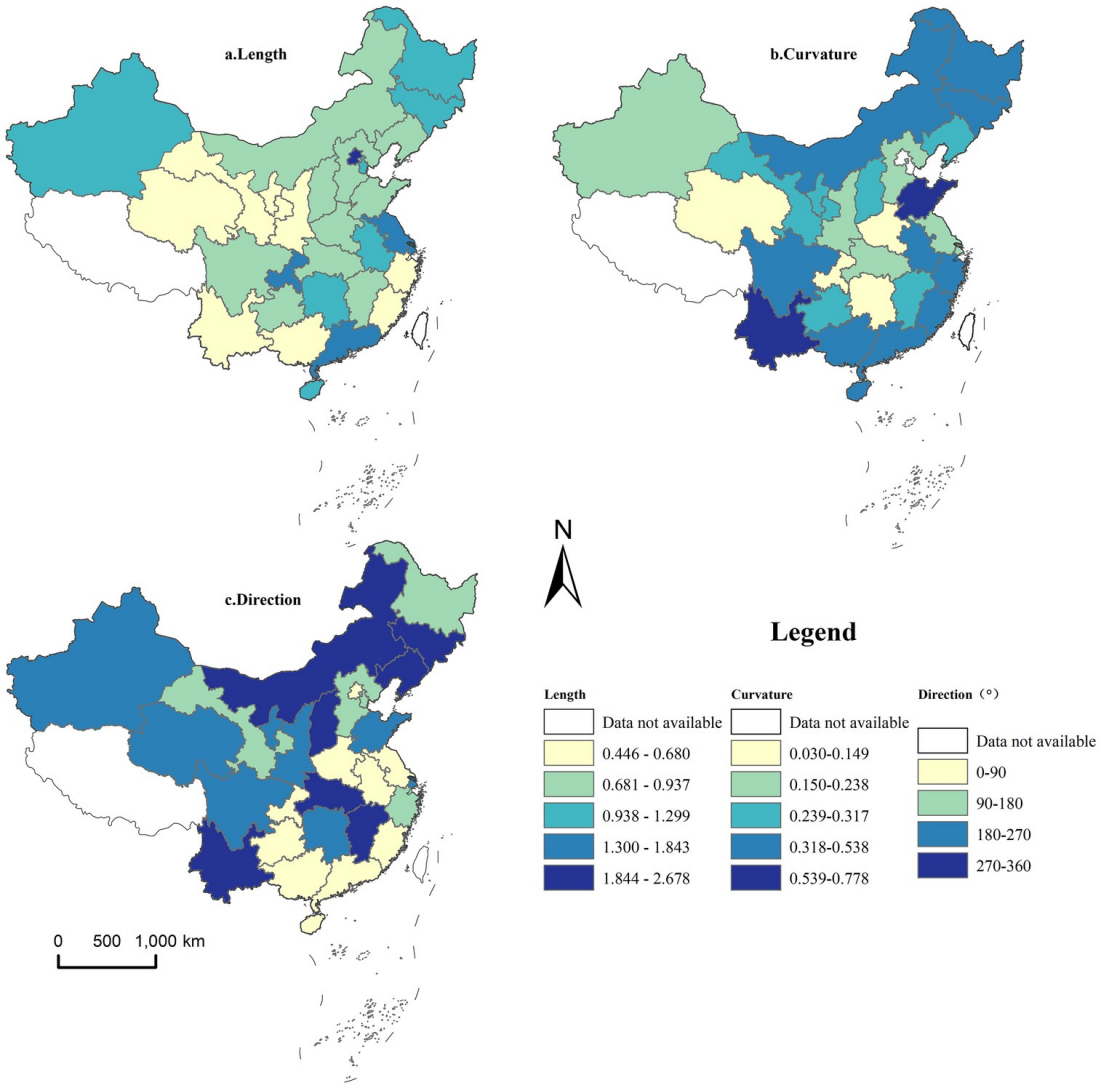
Spatial distribution of relative length, curvature, and movement direction of LISA space-time transition for rural land transfer in China.

Formulas (1) and (2) were employed to calculate the relative length and curvature of China’s rural land transfer ratio during the LISA space-time transition from 2005 to 2020. The ArcGIS software, utilizing the natural breakpoint method, was used for data classification and visualization. The outcomes are depicted in Fig 5.

Fig 5 reveals that regions with a high relative length of LISA space-time transition are primarily located in Beijing, Guangdong, Jiangsu, and Chongqing. Specifically, Beijing’s relative length is 2.678, significantly surpassing China’s average level of 1.000. This suggests a robust dynamic in the rural land transfer ratio for this area. This is intricately linked to the degree of local economic development, improvements in circulation assurance, positive response to the digital village strategy, and the advancement of the profound integration of the Internet with rural land circulation.

Conversely, areas exhibiting a low relative length are predominantly located in the western region and some eastern provinces, indicating a relatively modest dynamic in the rural land transfer ratio from 2005 to 2020. In total, 19 provinces, accounting for 63%, have a relative length of LISA space-time transition below China’s average level. This implies a relatively stable overall spatial pattern of rural land transfer in China from 2005 to 2020. This pattern primarily revolves around provinces characterized by high land productivity, well-established agricultural water conservancy facilities, robust rural transportation infrastructure, distinct farmers’ land concepts and family characteristics, diverse regional economic development levels, resource endowment conditions, and governmental policies, including laws and regulations.

The comprehensive LISA space-time transition curvature is merely 0.3208, affirming the outcomes of the preceding time path analysis. This indicates a robust lock-in effect in the spatial progression of China’s rural land transfer ratio from 2005 to 2020, signifying a relatively consistent spatial dependence and developmental trajectory. Regions with elevated curvature values, notably Shandong and Yunnan provinces, signify greater variability in rural land transfer compared to the local spatial dependence direction. This observation implies a deficiency in further endogenous motivation and sustainable development. Conversely, regions with low curvature, primarily concentrated in North China and some Western provinces, exhibit an average curvature of only 0.116, denoting the utmost stability in the spatial dependence direction.

#### LISA space-time transition moving direction analysis

By comparing the movement of each province in the Moran scatter plot at the beginning and end of the study period, we utilize formula (4) to calculate the directional shift in LISA space-time transition. This reveals the spatial integration characteristics in the local pattern evolution of geographical elements, as depicted in Fig 5c. The findings indicate that 18 provinces in China have witnessed synergistic increases in the rural land transfer ratio from 2005 to 2020, accounting for 60% of the total. This emphasizes the robust spatial integration in the evolution process of the research subject. Despite positive or negative changes, the evolution of China’s rural land transfer ratio during the research stage displays distinct homomorphic evolution characteristics. In specific directions, 10 provinces exhibit positive synergistic movement, constituting 33.33% of the total research units. These provinces are primarily located in the northwest, northeast, and north China areas, emphasizing the apparent synergistic growth effect in this region. At the same time, 8 provinces show negative co-movement, making up 27%, predominantly in specific areas of the central region and Zhejiang Province.

## Convergence analysis of regional differences in rural land transfer ratios in China

Analysis of the LISA space-time transition exposes a significant spatial disparity in the evolution trend of China’s rural land transfer ratio between 2005 and 2020. Will the disparities among provinces in China, and even within regions, gradually decrease over time? Is there a comparable convergence pattern among them? To address these inquiries, the initial examination involves analyzing the evolution trend of the standard deviation using σ convergence to determine the occurrence of convergence. Subsequently, the absolute β convergence test is applied to assess whether the disparities in land transfer ratios among provinces will gradually diminish over time. Finally, the club convergence test is conducted to ascertain whether each province’s land transfer ratio displays similar structural characteristics and if there are variations in convergence among different types of clubs.

### σ convergence analysis

**Fig 6:**
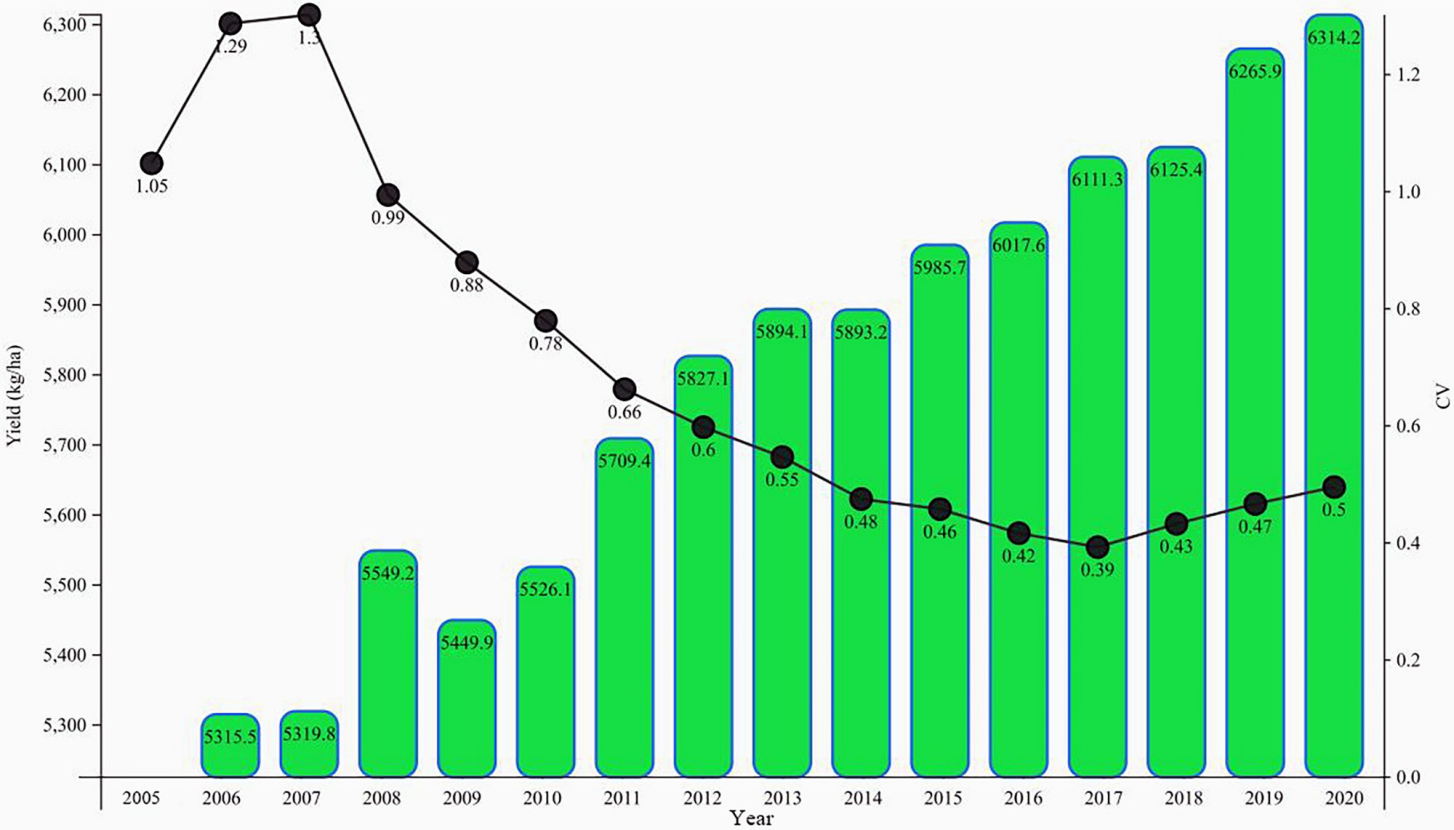
Evolution trend of the coefficient of variation in the rural land transfer ratio in China from 2005 to 2020.

Using Stata software, we computed the standard deviation and coefficient of variation (CV) for China’s rural land transfer ratio spanning 2005 to 2020. Fig 6 depicts a notable decrease in the coefficient of variation for China’s rural land transfer ratio. The evolution of the coefficient of variation from 2005 to 2020 can be broadly classified into three stages: 2005–2008, 2008–2017, and 2017–2020.

During the period from 2005 to 2008, a discernible inverted U-shaped trend emerged, characterized by an initial ascent followed by a descent. In this phase, China enacted the Rural Land Contracting Law to regulate land contracts. Despite some progress in land transfer policies, the actual situation remains cautious and constrained. Specifically, the policy environment for rural land transfer in China is imperfect, marked by numerous restrictions and obstacles to the transfer of land contract management rights. Local governments approach land transfer cautiously, expressing concerns about the social problems and income security of farmers after land loss. Simultaneously, the immature land transfer market restricts the scale and speed of land transfer in this period. Additionally, the lack of robust infrastructure hampers the development of agricultural industrialization and scale management. During that period, farmers lacked relevant knowledge and information, and were unaware of the risks and opportunities associated with land transfer. They maintained a conservative attitude toward land transfer, resulting in notable differences in transfer ratios.

Since 2008, there has been a steady decline in the coefficient of variation for China’s rural land transfer ratio. This trend is attributed to adjustments and support from government policies, the progress of agricultural industrialization, and enhancements in farmers’ education and awareness. Over this period, the Chinese government has enacted a series of land transfer policies. This signifies the initiation of institutionalization, proceduralization, and standardization in the management of rural land transfer in China. Concurrently, substantial transformations have transpired in China’s rural economic structure, characterized by an upswing in economic development levels in rural areas. This has, in turn, elevated farmers’ awareness and demand for land transfer, resulting in a gradual reduction in the disparity of land transfer ratios. Furthermore, policies and funding have supported the development and expansion of farmers’ cooperatives and agricultural enterprises. Through participation in the operations of these entities via land transfer, farmers have secured more stable income and employment opportunities. Additionally, there has been a general improvement in the education level of Chinese farmers, resulting in increased awareness and understanding of land transfer. Farmers are now more willing to engage in land transfer activities, paying closer attention to this aspect and recognizing the economic benefits and opportunities associated with it. Consequently, post-2008, the coefficient of variation for China’s rural land transfer ratio has exhibited a fluctuating downward trend, signifying the diminishing regional differences and the flourishing state of land transfer activities.

After 2017, influenced by factors like disparities in economic development and local policy adjustments, the coefficient of variation for the land transfer ratio exhibited an upward trend. Amid the comprehensive reforms in agriculture, rural areas, and the farming sector in China, agricultural land transfer is encountering new opportunities for development. Consequently, the overall coefficient of variation shows a fluctuating downward trend. Toward the end of the research period, there was a slight upward trend, although not significant. The trend is converging, yet with the ongoing progress of land transfer efforts, fundamental conditions differ across regions. Local governments formulate appropriate measures for managing the scale of cultivated land. This will prevent a one-sided pursuit of speed and scale growth, ensuring sustainable land use and the stable development of rural society. As various regions implement a series of policies and economic development levels improve, the land transfer ratio in each province will continue to rise, reducing differences. Overall, there is σ convergence in China’s rural land transfer ratio.

### Absolute β convergence analysis

Using Formula (6), this study evaluates the absolute β convergence of China’s rural land transfer ratio across different stages from 2005 to 2020. Due to the provincial panel data foundation of this research, the fixed-effect model is selected for the β convergence model. The results are displayed in Table 2.

**Table 2:** The Absolute β convergence test of China’s rural land transfer ratio from 2005 to 2020.

Period	Constant	Coefficientβ	F	Convergence	Convergence rate θ
**2005–2020**	0.0350***	-0.0645***	27.02	√	0.0042
	10.8	-5.2			
**2005–2010**	0.0310***	-0.1004	2.05	√	0.0066
	4.34	-1.43			
**2010–2015**	0.0577***	-0.1022***	9.79	√	0.0067
	6.6	-3.13			
**2015–2020**	0.2506***	-0.6711***	35.85	√	0.0695
	5.92	-5.99			

Note:

*p < 0.1,

**p < 0.05,

***p < 0.01,

the t-values are in parentheses.

The β coefficients for China’s rural land transfer ratio during the periods 2005–2010, 2010–2015, and 2015–2020 are notably significant and negative. This indicates a distinct absolute β convergence in China’s rural land transfer ratio throughout each research stage. The convergence rate shows an upward trend over the three periods, increasing from 0.0066 in 2005–2010 to 0.0067 in 2010–2015, and further rising to 0.0695 in 2015–2020. In summary, China’s rural land transfer ratio demonstrates absolute β convergence, signifying that provinces with higher land transfer rates exert a "trickle-down effect" on provinces with lower land transfer rates, enabling the latter to narrow the speed gap of land transfer.

Secondly, the report of the Nineteenth National Congress introduced the rural revitalization strategy, with land reform as a pivotal component. The implementation of this strategy has elevated economic development in rural areas, enticing more farmers to participate in land transfer. Moreover, it has spurred rural economic development through improvements in infrastructure, the promotion of agricultural modernization, and the development of rural industries. Recently, farmers have shown heightened awareness and understanding of the benefits of land transfer, leading to increased willingness to participate in the process. Recently, Chinese agriculture has accelerated industrialization, fostering the scale, specialization, and marketization of agricultural production. Consequently, farmers have transferred their land to large-scale agricultural enterprises or professional cooperatives to achieve improved economic benefits and stable income. Concurrently, as the gap between urban and rural economic development narrows, farmers’ comprehension of land transfer has progressively expanded. Currently, farmers show a greater willingness to transfer their land through leasing or cooperative operations, aiming for a stable income, risk reduction in agricultural operations, and exploration of opportunities in other industries. Furthermore, with the progress and widespread adoption of information technology, rural areas have undergone increased informatization. This facilitates farmers’ access to relevant information regarding agricultural production and land transfer. This enhanced transparency in information has proven beneficial, mitigating uncertainty and risk in the land transfer process, thereby making it more convenient and secure.

### Club convergence analysis

China’s provinces are classified into distinct regions or "clubs" based on their economic development level to investigate the presence of club convergence in the country’s rural land transfer ratio. The results, presented in Table 3 and modeled through a fixed-effect model, show that the β coefficients of the eastern, western, and central regions are all negative and pass the 5% significance level test. This indicates the presence of club convergence in land transfer across all regions.

**Table 3:** Convergence test of China’s rural land transfer club from 2005 to 2020.

Region	Constant	Coefficient β	F	Convergence	Convergence rate θ
Eastern	0.0441***	-0.0691***	8.87	√	0.0045
	5.91	-2.98			
Western	0.0286***	-0.0856***	17.69	√	0.0056
	7.35	-4.21			
Central	0.0335***	-0.0455**	5.79	√	0.0029
	6.55	-2.41			
National	0.0350**	-0.0645***	27.02	√	0.0042
	10.8	-5.2			

Note:

*p < 0.1,

**p < 0.05,

***p < 0.01,

the t-values are in parentheses.

The convergence rates for the eastern and western regions are 0.0045 and 0.0056, respectively, exceeding the overall convergence rate for China. Conversely, the central region shows a convergence rate of 0.0029, slightly below the overall convergence rate for China. However, overall, the convergence rates for the eastern, western, and central regions are relatively modest, similar to the overall convergence rate for China. This suggests a reduction in disparities in rural land transfer among different provinces in China. Several factors contribute to this, including the enactment of a series of land policies, the rural revitalization strategy, the advancement of agricultural industrialization, shifts in farmers’ consciousness, and the promotion of technological progress and informatization. These factors have positively influenced the promotion of convergence. Furthermore, with the ongoing progress in China’s land transfer efforts, the land transfer model has undergone innovation, transitioning gradually toward a market-oriented and diversified approach. The cumulative impact of these factors has standardized and elevated the overall quality of land transfer. Consequently, the gap in rural land transfer ratios among the eastern, western, and central regions has reduced, with a minor disparity in convergence speed.

## Analysis of the driving factors of the evolution of rural land transfer ratio in China

### GTWR estimation results

Estimating the GTWR model requires the absence of multicollinearity among explanatory variables. Stata 16.1 is used in this study to evaluate the multicollinearity of the model. The results indicate that the variance inflation factor (VIF) for all explanatory variables is below 10, and the average VIF is also below 10 (As shown in Table 4). This finding indicates the absence of multicollinearity, meeting the prerequisites for estimating the GTWR.

**Table 4:** VIF coefficient test of influencing factors on land transfer ratio difference.

Variable	VIF	1/VIF
LTR	—	—
f1	1.460	0.684
f2	1.30	0.771
f3	1.340	0.747
f4	1.420	0.704
f5	2.010	0.499
f6	1.420	0.705
VIF	mean value	1.49

Note:

LTR means land transfer ratio,

f1 means land productiv ity,

f2 means per capita mechanical power,

f3 means total highway mileage,

f4 means per capita income of farmers,

f5 means irrigation guarantee rate,

f6 means the proportion of agricultural output value.

Additionally, ArcGIS 10.8 software is utilized to analyze OLS, GTWR, GWR, and TWR models, with R_2_ and AIC chosen as evaluation indices for the models. The criteria state that a higher R_2_ is preferable, and a lower AICc is more favorable. The results, shown in Table 5, indicate that the GTWR model displays a higher overall R_2_ and a lower AICc. Consequently, the GTWR model is employed in this study to elucidate the factors influencing regional disparities in China’s rural land transfer ratio.

**Table 5:** Related parameters of GTWR.

Parameter	Bandwidth	ResidualSquares	AICc	R^2^	AdjustedR^2^	Spatio-temporalDistanceRatio
**value**	0.114996	1.19241	-1308.25	0.922051	0.921062	0.541833

Based on relevant research findings [5, 36–42], the land transfer ratio is chosen as the dependent variable, and six indicators are selected to construct the GTWR model. The selected indicators include land productivity, per capita total mechanical power, total highway mileage, per capita net income of farmers, irrigation guarantee rate, and agricultural output value. The parameters of the GTWR model for each variable influencing the spatial and temporal evolution of China’s rural land transfer ratio production from 2005 to 2020 were obtained through the AICc information criterion method (As shown in Table 4). The goodness-of-fit analysis reveals that both R_2_ and corrected R_2_ exceed 90%, indicating the effectiveness of the entire GTWR estimation model in explaining the impact of each variable on the land transfer ratio. This suggests that the conclusions derived from the GTWR model are more reasonable.

### The evolution of spatial distribution of driving factors’ influence

**Fig 7:**
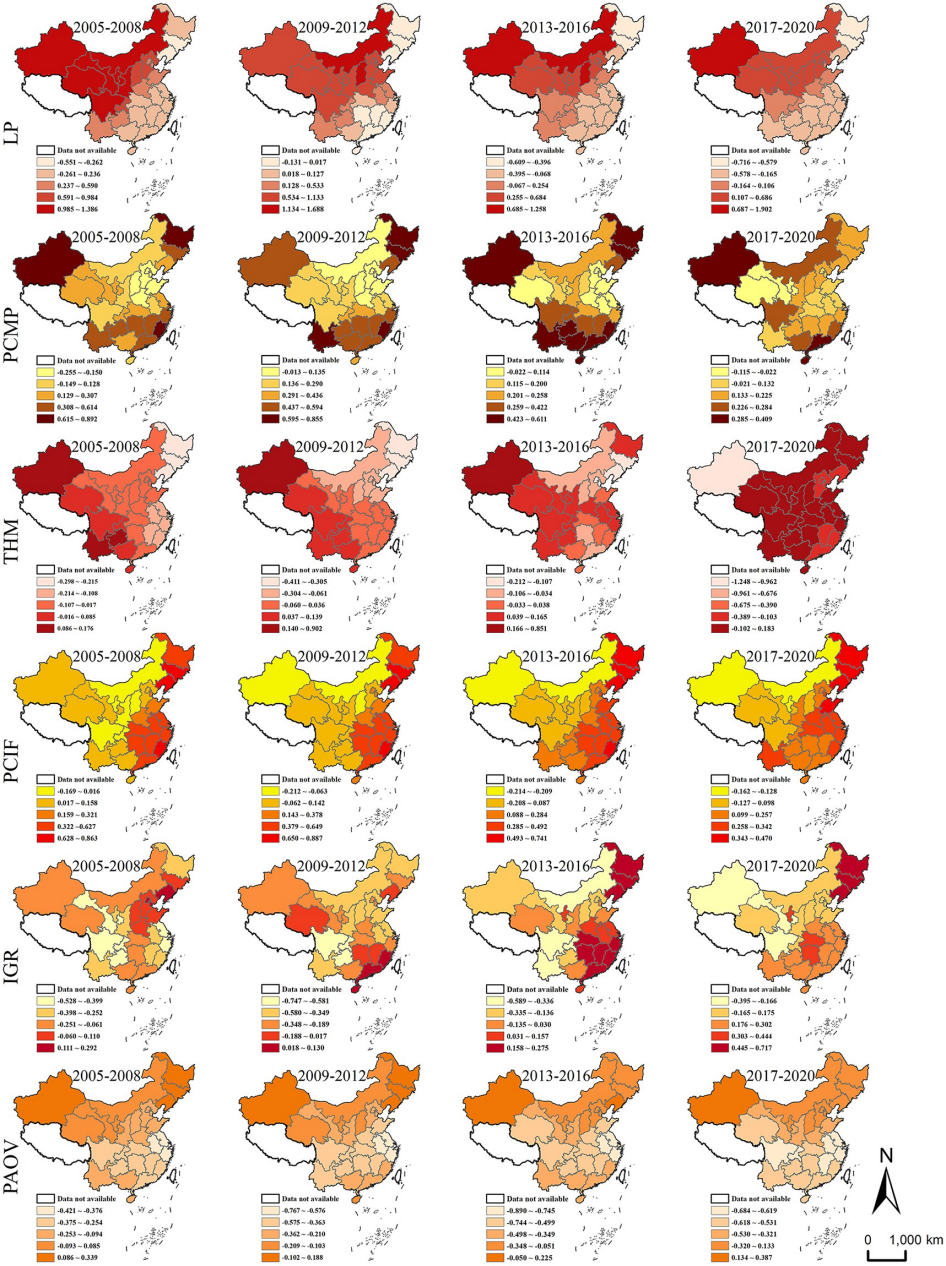
Spatial distribution of regression coefficients in GTWR model.

Spatial variations exist in the influence of distinct driving factors on the growth rate of land transfer across different temporal intervals (As shown in Fig 7).

#### Land productivity

Land productivity serves as an indicator of land production capacity, commonly measured by the number of products or the value of output per unit of land area over a specific time period [36]. An interaction effect occurs between land productivity and land transfer. The impact of cropland productivity on land transfer includes the power, mode, and scale of the transfer. High-productivity land, on one hand, can yield increased agricultural returns and is more likely to attract participation in land transfer by large-scale agricultural enterprises or investors. On the other hand, low-productivity land may be more readily transferred, as farmers might find it more advantageous to lease or sell. Regression results indicate that the impact of land productivity on land transfer is both positive and negative from 2005 to 2020. Overall, the impact of land productivity on the growth rate of land transfer is progressively increasing. Spatially, positive high-value areas are concentrated in western China from 2005 to 2020, with a relatively stable spatial pattern. Conversely, negative high-value areas are primarily found in the central and eastern regions. In the central region, the provinces of Jilin and Heilongjiang exhibit lower absolute values of regression coefficients, indicating a relatively stable spatial pattern. Since 2005, no fundamental changes have occurred, indicating that the contribution of land productivity to land transfer in Northeast China is low and even exhibits a downward trend

#### Per capita mechanical power

The increase in per capita mechanical power input significantly affects land transfer. Firstly, the utilization of agricultural machinery maximizes land benefits, facilitates rural labor force transfer, and expedites land transfer. Secondly, agricultural mechanization is crucial for reducing costs and enhancing efficiency in agricultural production [37]. Consequently, it significantly improves overall agricultural production efficiency. Regression results show a spatial distribution pattern in the regression coefficient of per capita total mechanical power and land transfer between 2005 and 2020, with higher values in the southern regions and lower values in the northern regions. Hotspots, such as Xinjiang, Northeast China, and Fujian, show a diffusion trend from Fujian to surrounding provinces. Conversely, the cold spot area demonstrates a diffusion trend from the North China Plain to surrounding provinces. Between 2017 and 2020, the number of hotspots began to decrease. This suggests that the impact of per capita mechanical power on the growth rate of land transfer is gradually diminishing. This is because, in line with the law of diminishing marginal returns of land, the impact of per capita mechanical power on land transfer gradually increases as PCMP rises. After reaching a certain threshold, the impact peaks. Beyond this limit, the impact of per capita mechanical power on land transfer diminishes as agricultural machinery power improves.

#### Total highway mileage

Total highway mileage is a commonly used metric to assess the scale and extent of transportation infrastructure, evaluating the transportation network of a country or region. Firstly, transportation infrastructure is crucial for the development of China’s agricultural production. Secondly, highways are fundamental for ensuring the industrialization of agriculture. The ongoing improvement of transportation infrastructure, including highways and bridges, influences the behavioral decisions of both the supply and demand sides of land transfer, thereby affecting land transfer [38]. Based on the regression results, the regression coefficient showed a spatial trend of gradual decrease from west to east during the period 2005 to 2016. This suggests that since 2005, the driving impact of total highway mileage on land transfer in the western region has been increasing. Nevertheless, the majority of cold spots in China’s total highway mileage regression coefficient are concentrated in the provinces of Northeast China, North China, and Southeast China. After 2017, the influence of total highway mileage on the growth rate of land transfer gradually weakened. This is because, following the law of diminishing marginal returns of land, the influence of total highway mileage gradually increases as THM rises. After reaching a certain threshold, this influence peaks. Beyond this limit, the influence of total highway mileage on land transfer decreases as highway mileage increases.

#### Per capita income of farmers

Per capita income of farmers refers to the total income of rural households in a given year, net of expenses. Firstly, farmers’ household income is a crucial factor that both motivates and constrains land transfer. It significantly influences farmers’ decision-making processes regarding land transfer. Secondly, serving as an indicator of available resources, production, and lifestyle, farmers’ household income has become an internal driving force in the land transfer process [39]. Based on regression results, the impact of per capita income of farmers (PCIF) on land transfer has remained stable from 2005 to 2020. Regions with high positive values were concentrated in the northeast, the Yangtze River Delta, and the southeast coastal areas, suggesting a significant influence of PCIF on land transfer in these regions. Conversely, regions with low negative values, such as Xinjiang and Inner Mongolia, showed a weak influence of PCIF on land transfer in these areas.

#### Irrigation guarantee rate

The construction of water conservancy infrastructure for farmland is of pivotal significance in the context of rural water conservancy development, constituting a fundamental underpinning for the advancement of sustainable agriculture [40]. Moreover, it plays a crucial role in ensuring the long-term stability of rural areas. Irrigation facilities, as essential components of agricultural infrastructure, significantly facilitate land transfer. On one hand, the construction of water conservancy infrastructure for farmland and land transfer is mutually complementary. The advancement of water conservancy construction for farmland promotes simultaneous progress in land transfer. On the other hand, policies related to land transfer actively encourage the construction of water conservancy facilities for farmland. Regression results reveal a growing influence of Inter-Governmental Relations (IGR) on land transfer, indicating an escalating driving effect of IGR on land transfer since 2005. Regions with elevated positive values show a gradual concentration from North China through Northeast China, the Yangtze River Delta, to the Southeast coastal provinces. Conversely, regions with markedly low negative values predominantly concentrate in most western provinces. This observation signifies a significantly higher reliance on Inter-Governmental Relations (IGR) in the eastern region compared to the western region. This discrepancy arises from the fact that, in the development of rural infrastructure in China, the expansion of agricultural irrigation areas is notably limited, with certain regions.

#### The proportion of agricultural output value

The total output value of agriculture represents the monetary value of all products from agriculture, forestry, animal husbandry, and fisheries generated within a defined timeframe, typically one year. The proportion of agricultural output value represents the ratio of the entire agricultural output value to the total economic output value. This metric reflects the comprehensive scale and magnitude of regional agricultural production [41]. A higher proportion of agricultural output value is crucial for facilitating land transfer processes and promoting large-scale agricultural operations, resulting in greater benefits in refining land management policies and enhancing land transfer mechanisms. Based on regression findings, the influence of Proportion of Agricultural Output Value (PAOV) on land transfer from 2005 to 2020 has remained stable. Analyzing the spatial distribution changes in the regression coefficient, PAOV shows a geographical pattern characterized by higher values in the northern regions and lower values in the southern regions. This pattern has shown relative stability since 2005. Regions with elevated PAOV regression coefficients consistently cluster in the northwest and northeast, indicating a robust correlation between land transfer and PAOV in these areas. In contrast, regions with lower PAOV regression coefficients remain stable in the central and southern areas, showing no notable changes from 2005 to 2020. This implies that PAOV has a limited influence on land transfer in southern China.

## Conclusions and discussions

### Main conclusions

Land transfer significantly contributes to rural revitalization, playing a crucial role in promoting economic development, augmenting farmers’ income, and optimizing land resource allocation in China. This study investigates the spatial distribution characteristics, pattern evolution, and convergence of China’s land transfer ratio across 30 provinces (excluding Tibet and Taiwan) from 2005 to 2020. The Geographically Weighted Regression (GTWR) model was employed to identify the driving forces behind these trends.

(1) The land transfer ratio in China has exhibited a fluctuating upward trend, demonstrating an increasing pattern from west to east and from north to south. The primary spatial disparity exists between the eastern and western regions, with a relatively minor difference between the northern and southern regions. Post-2020, it is anticipated that the disparities between the northern and southern regions, as well as between the eastern and western regions, will achieve greater balance.
(2) The LISA space-time transition unveils a pronounced spatial locking effect in China’s rural land transfer, with its evolution showcasing resilient spatial integration. The relative length implies a relatively stable overall spatial pattern, and the curvature indicates consistent developmental direction with spatial dependence. The moving direction signifies stable spatial co-growth.
(3) Between 2005 and 2020, substantial σ convergence, absolute β convergence, and club convergence are observed in China’s rural land transfer ratio. This indicates that internal differences in land transfer ratios among provinces and regions are gradually diminishing, with higher land transfer ratios in specific areas influencing the surrounding lower areas. This diffusion enables lower regions to catch up with the land transfer gap. Concurrently, the convergence speed difference in the eastern, western, and central regions is small, reflecting the overall convergence speed of China. This suggests a diminishing disparity in rural land transfer levels among provinces.
(4) Factors such as transportation infrastructure, farmland water conservancy construction, and the economy collectively influence the spatial and temporal evolution pattern of China’s rural land transfer ratio. Over time, the dominant driving force shifts from land productivity and per capita total mechanical power to the irrigation guarantee rate and total highway mileage. This suggests that transportation infrastructure and agricultural infrastructure are playing an increasingly significant role in enhancing the land transfer ratio.

### Discussion

Amid China’s ongoing and swift agricultural modernization, rural land transfer is intricately linked to the substantive rights and interests of millions of Chinese citizens. The rural land transfer ratio in China displays fluctuations and an overall increase, with rapid progress observed in specific regions. Although high-value rural land transfer ratios are still predominantly concentrated in the central and eastern regions of China, there has been a gradual shift, and certain provinces in the western region are emerging as areas with medium-high value transfer ratios. It is essential to note that the land transfer ratio in the western region remains comparatively lower than that in the eastern region.

The western region experiences relatively lower economic development, and the degree of marketization in land transfer differs from that in the eastern region. The level of land transfer correlates closely with the presence of agricultural water conservancy facilities and transportation infrastructure in rural areas of the western region. The foundational strength of agricultural water conservancy facilities in the western region is relatively weak, with certain deficiencies in construction technology. The management technology of water conservancy facilities is outdated, and the transportation infrastructure is relatively insufficient.

The primary challenge in rural land transfer arises from the unclear relationship between land property rights and the absence of government regulation. Consequently, addressing these issues requires collaboration between the government and all societal stakeholders. On the one hand, it is imperative to elucidate land property rights, enhance the social insurance system associated with land transfer models, enact pertinent laws and regulations, and establish a legal framework for land transfer. Concurrently, there is a need to cultivate and standardize the rural land market, fortify the administration of the rural land market, mitigate risks for farmers involved in land transfer, and comprehensively safeguard the interests of farmers. On the other hand, the government should tailor its approach to local circumstances, align with the economic development trends across diverse regions, steer the centralized development of rural land, facilitate systematic and judicious transfers, and encourage the infusion of capital, technology, and other pivotal factors into rural areas. This aims to refine the mechanism of rural land transfer in China, propel the establishment of agricultural industrial parks characterized by economies of scale, foster prosperity in rural industries, and contribute to the advancement of China’s new urbanization.

This study employs long-time series data from diverse Chinese provinces, primarily for macro-scale analysis. Nevertheless, there are limitations in expressing findings at the micro-scale. Owing to constraints in data acquisition, there exists ample opportunity for further enhancement and refinement in selecting indicators pertinent to the influencing factors of rural land transfer in China. Additionally, owing to space constraints, a detailed analysis of the future development trend of China’s land transfer rate is not feasible. China holds a significant position as a global agricultural producer and trading nation. A pivotal goal in the modernization of Chinese agriculture is expediting the shift from an agricultural powerhouse to one aligned with China’s national conditions. Consequently, research on the prospective development of rural land transfer in China will concentrate on two primary aspects. First, investigating the integration of agricultural land transfer with the establishment of high-standard farmland to propel agriculture towards scale, specialization, and industrialization. The second aspect involves examining strategies to facilitate the organic fusion of Internet technology and agricultural land transfer, thereby augmenting the competitiveness of China’s agriculture.

## Supporting information

S1 Table The operational results of GTWR model. (XLSX)

S2 Table Statistics on relevant variables influencing the rural land transfer ratio in China. (XLSX)

S3 Table The absolute β convergence test of China’s rural land transfer ratio from 2005 to 2020. (XLSX)

S4 Table Convergence test of China’s rural land transfer club from 2005 to 2020. (XLSX)

S5 Table VIF coefficient test of influencing factors on land transfer ratio difference. (XLSX)

S6 Table Related parameters of GTWR model. (XLSX)
